# Supplementary material for: A tighter constraint on Earth-system sensitivity from long-term temperature and carbon-cycle observations
Source: Nat Commun. 2021 May 26;12:3173. doi: 10.1038/s41467-021-23543-9 (PMC8154887; doi:10.1038/s41467-021-23543-9)
Supplement: Supplementary file 1 — Supplementary Information [file 41467_2021_23543_MOESM1_ESM.pdf]

Supplementary Information

Accompanying "A tighter constraint on Earth-system sensitivity from long-term temperature and carbon-cycle observations", by Tony E. Wong, Ying Cui, Dana L. Royer and Klaus Keller

This supplement contains figures supporting the results and discussion presented in the main text. Specifically, we present:

- additional plots showing the 5-95% probability ranges and best estimates of the time series parameters (Supplementary Figure 1) and the autocorrelation functions for each time series parameter (Supplementary Figure 2);
- the relationship between the proxy data CO_2 concentrations and the width of the uncertainty range for each data point (Supplementary Figure 3);
- the distributions of estimated Earth-system sensitivity parameter (ΔT_{2x}) in the control experiments, as well as the results of Krissansen-Totton and Catling (2017)[1] and the sensitivity experiment in which the model simulations are forced into agreement with the Cretaceous temperatures at 90 Myr ago (Supplementary Figure 4);
- the a posteriori mean time series parameters that are most affected in the Cretaceous temperature-matching experiment, relative to the original set of experiments (Supplementary Figure 5);
- a sensitivity experiment in which a linear change in ΔT_{2x} is assumed instead of the step function change in the default GEOCARB configuration (Supplementary Figure 6);
- a hypothetical likelihood surface in which a skew-normal mixture model is fit to the data within each 10 Myr model time step (Supplementary Figure 7);
- the raw CO_2 concentration data from Foster et al. (2017)[2] and the fitted precalibration windows used in the main text (Supplementary Figure 8); and
- boxplots for the distributions of ΔT_{2x} for sub-sample sizes ranging from 1,000 to 10,000 (full sample) (Supplementary Figure 9).

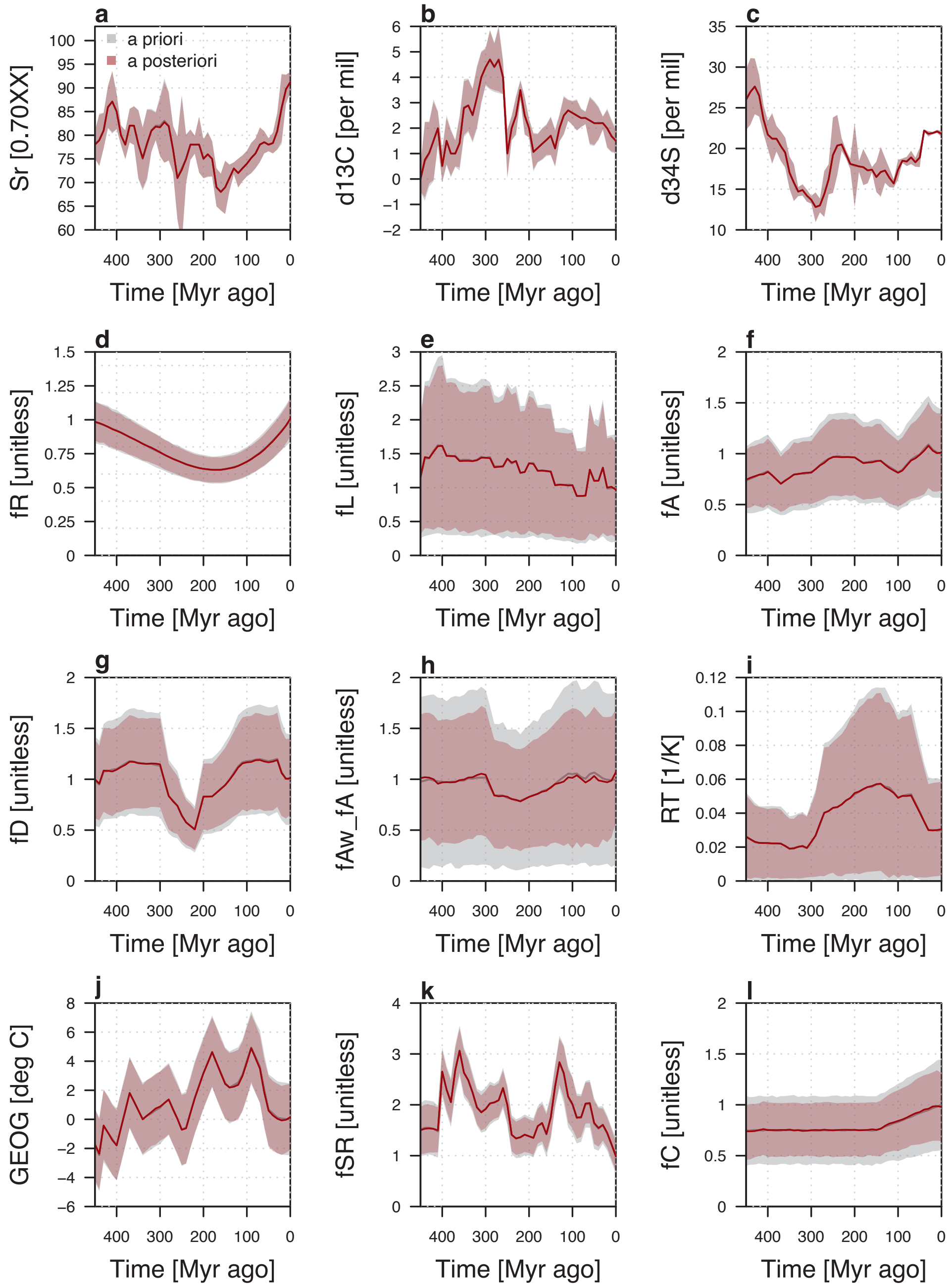

Supplementary Figure 1. Time series GEOCARB input parameters prior ranges (gray shaded region), precalibration results for the 95% credible range (red shaded region) and median (solid red lines). (a) isotope ratio $^{87}Sr/^{86}Sr$ of shallow-marine carbonate, where the values given here are the third and fourth decimal places of the ratios, (b) isotope ratio $\delta^{13}C$ of shallow-marine carbonate, (c) isotope ratio $\delta^{34}S$ of marine sulfate, (d) f_R, the effect of continental relief on chemical weathering rates, relative to present-day, (e) f_L, the fraction of total land area covered by carbonates, relative to present-day, (f) f_A, the land area relative to present-day, (g) f_D, the global river runoff relative to present-day, (h) f_{AW}/f_A, the fraction of total land area that undergoes chemical weathering, relative to present-day, (i) RT, a coefficient modulating how changes in temperature affect the runoff rate, (j) GEOG, the change in global surface temperature relative to present-day, assuming present-day CO_2 and solar luminosity, (k) f_{SR}, the rate of seafloor spreading relative to present-day, and (l) f_C, the effect of carbonate sediments in subducting oceanic crust on CO_2 degassing rate.

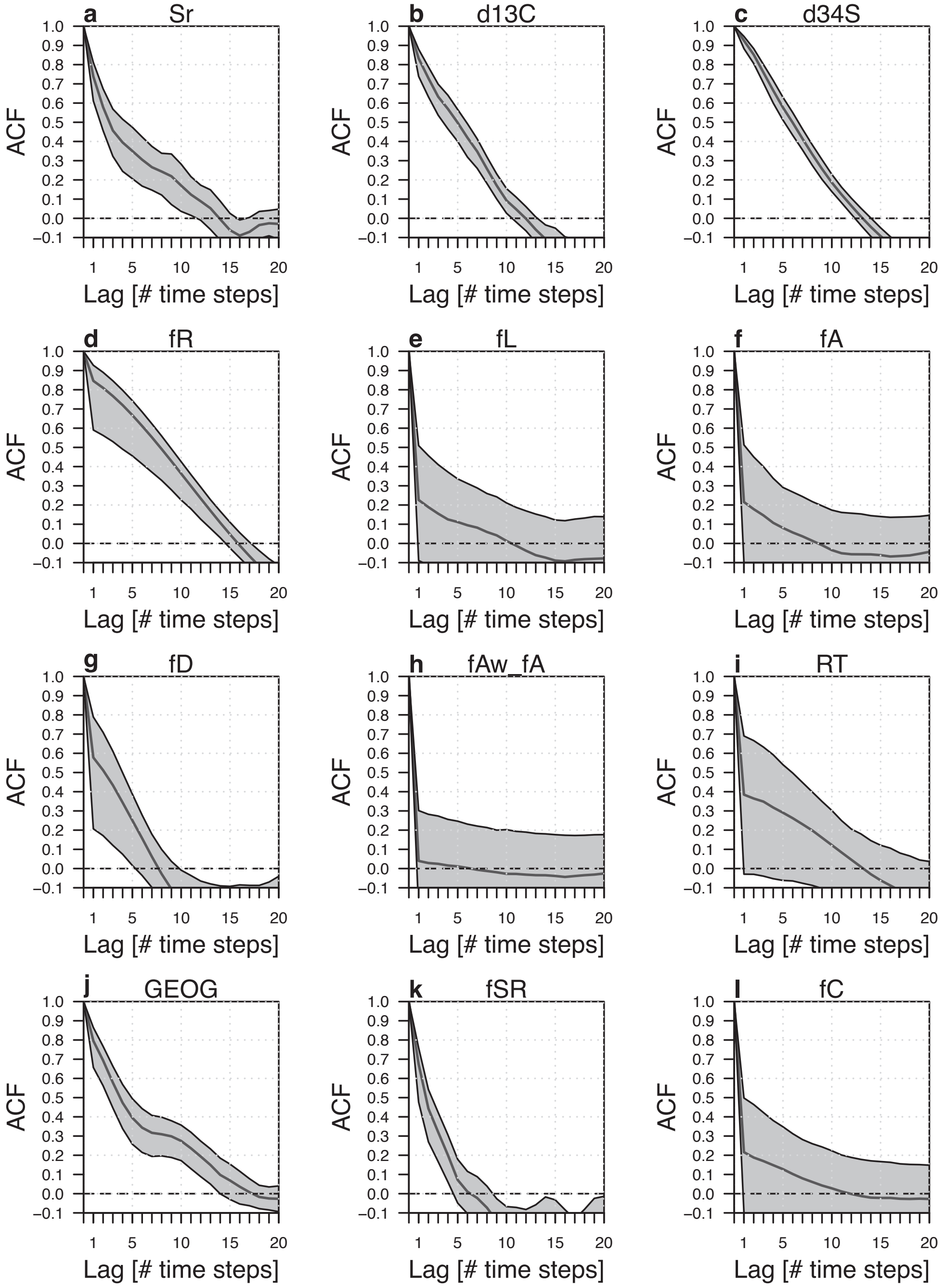

Supplementary Figure 2. Autocorrelation function for the time series parameters from the ensemble using both CO_2 and temperature data and a %outbound threshold of 30%. The solid black central lines denote the ensemble median and the gray shaded ranges denote the 95% credible range. The 12 time series parameters shown are: (a) isotope ratio $^{87}Sr/^{86}Sr$ of shallow-marine carbonate, (b) isotope ratio $\delta^{13}C$ of shallow-marine carbonate, (c) isotope ratio $\delta^{34}S$ of marine sulfate, (d) f_R, the effect of continental relief on chemical weathering rates, (e) f_L, the fraction of total land area covered by carbonates, (f) f_A, the land area, (g) f_D, the global river runoff, (h) f_{AW}/f_A, the fraction of total land area that undergoes chemical weathering, (i) RT, a coefficient modulating how changes in temperature affect the runoff rate, (j) GEOG, the change in global surface temperature, assuming present-day CO_2 and solar luminosity, (k) f_{SR}, the rate of seafloor spreading, and (l) f_C, the effect of carbonate sediments in subducting oceanic crust on CO_2 degassing rate.

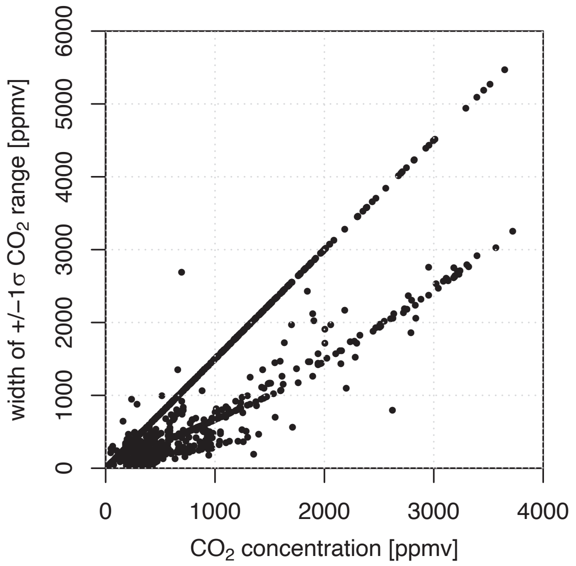

Supplementary Figure 3. Relationship between CO_2 concentration and uncertainty. The uncertainty (vertical axis) is measured by the difference between the high and low uncertainty estimates for each CO_2 proxy data point given by ref. 2.

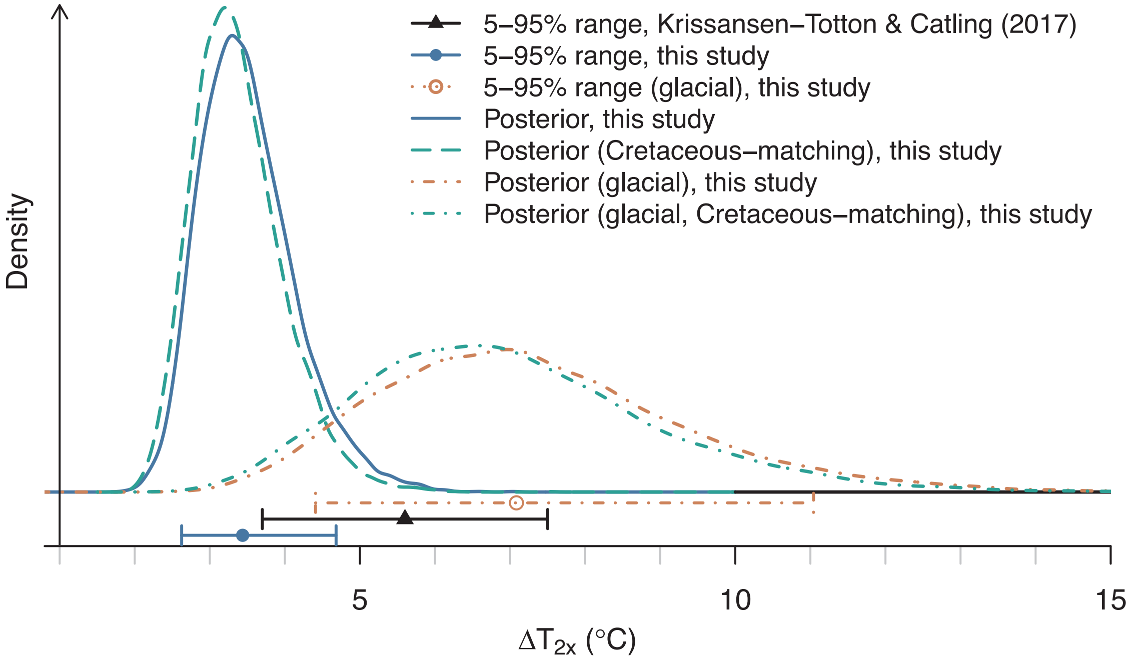

Supplementary Figure 4. Results for ESS parameter ΔT_{2x} from main text with a %outbound threshold of 30% (solid blue line and filled circle), the corresponding glacial period ΔT_{2x} (orange dot-dashed line and open circle), the ΔT_{2x} distribution from the Cretaceous temperature-matching experiment, and the 5-95% probability range for ΔT_{2x} reported by ref. 1 (black range and filled triangle).

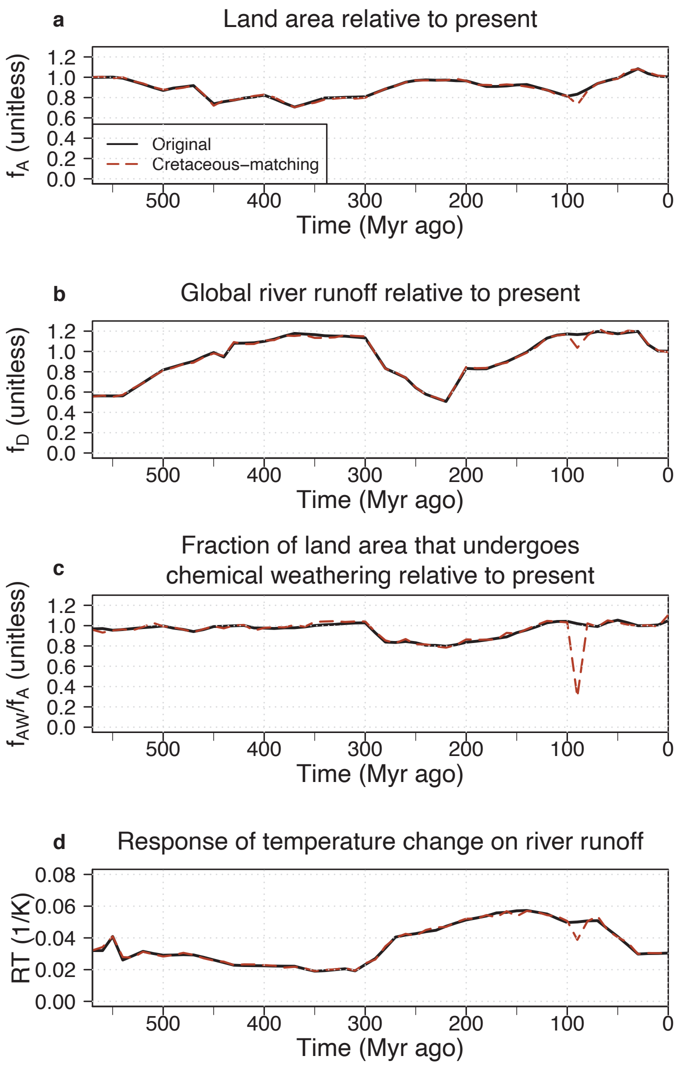

Supplementary Figure 5. A posteriori means for the most affected time series parameters from the original (solid black line) and Cretaceous-matching (dashed red line) experiments. Shown are the time series for (a) the land area relative to present, (b) the global river runoff relative to present, (c) the fraction of land area that undergoes chemical weathering relative to present, and (d) the response of temperature change on river runoff.

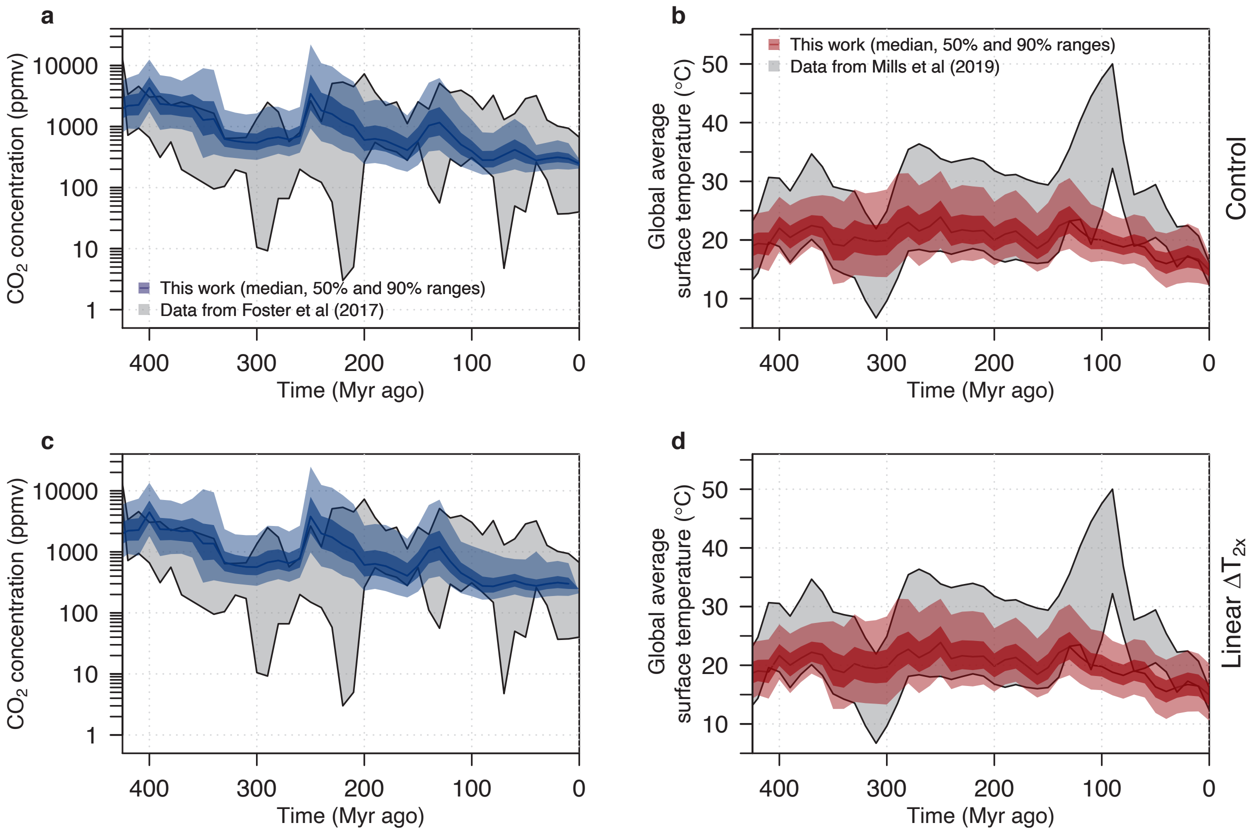

Supplementary Figure 6. Model hindcast, using both CO_2 and temperature data for precalibration and a %outbound threshold of 50% (shaded regions). The gray shaded regions show the data compilations for CO_2[2] and temperature[3]. The light colored shaded regions denote the 90% probability range from the precalibrated ensemble, the dark shading denotes the 50% probability range and the solid colored lines show the ensemble medians. The top row (a, b) corresponds to the control experiment (analogous to Fig. 3); the bottom row (c, d) corresponds to the experiment where the Earth system sensitivity parameter undergoes a linear change from its nonglacial value to its glacial value between 130 and 40 Myr ago.

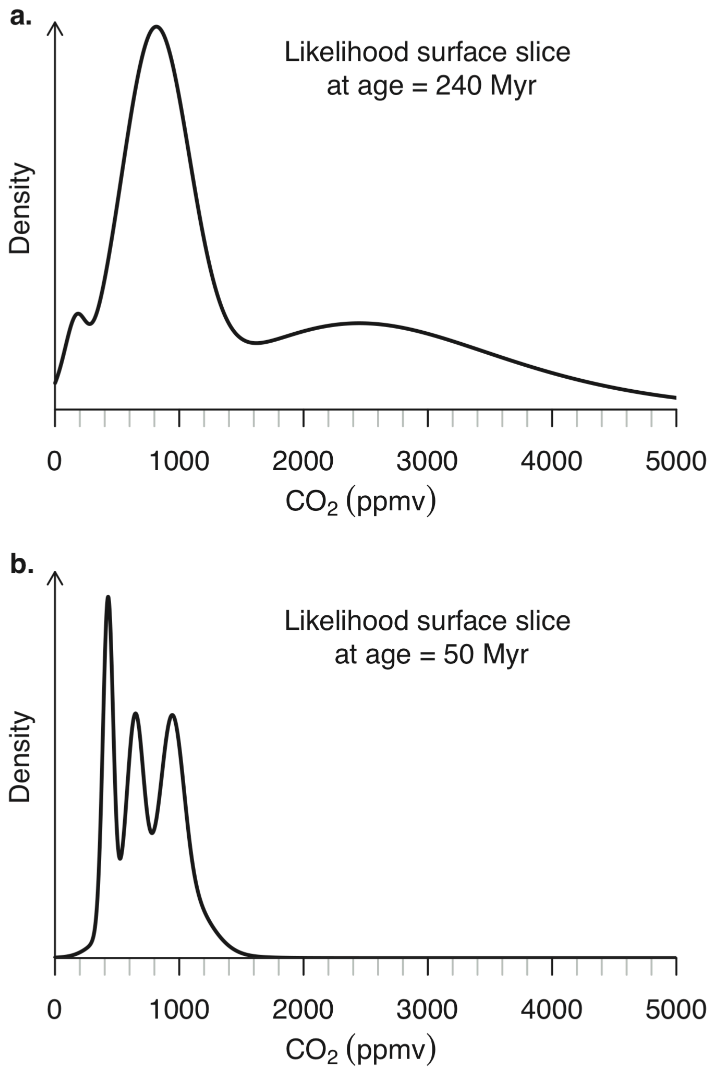

Supplementary Figure 7. Time slices of a skew-normal mixture model likelihood surface at ages 240 Myr (a) and 50 Myr (b).

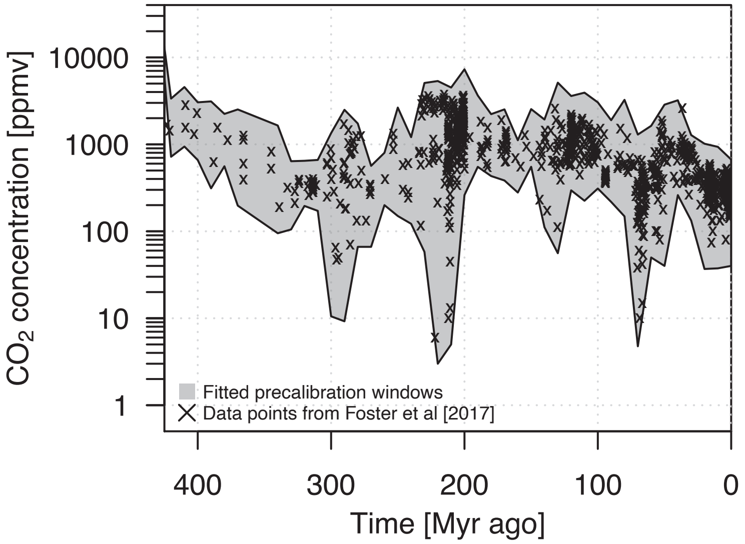

Supplementary Figure 8. CO_2 proxy data points[2] (x) and the fitted precalibration windows (gray shaded region).

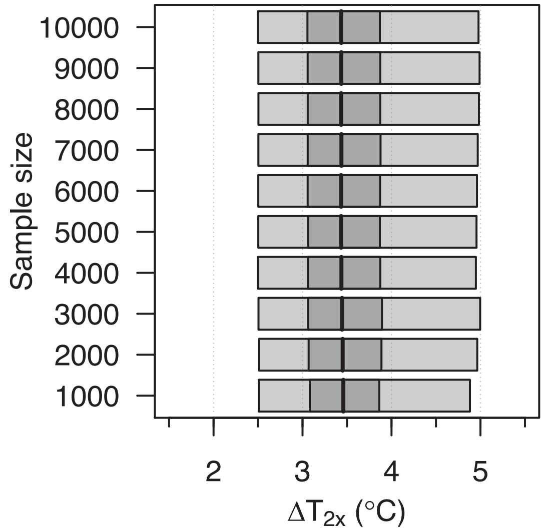

Supplementary Figure 9. Estimated 95% (light shading) and 50% (dark shading) credible ranges and medians for Earth-system sensitivity parameter (ΔT_{2x}) from the experiments presented in the main text for sample sizes ranging from 1,000 to 10,000 in increments of 1,000.

Supplementary References

1. Krissansen-Totton, J. & Catling, D. C. Constraining climate sensitivity and continental versus seafloor weathering using an inverse geological carbon cycle model. *Nat. Commun.* **8**, (2017).
2. Foster, G. L., Royer, D. L. & Lunt, D. J. Future climate forcing potentially without precedent in the last 420 million years. *Nat. Commun.* **8**, (2017).
3. Mills, B. J. W. *et al.* Modelling the long-term carbon cycle, atmospheric CO_2, and Earth surface temperature from late Neoproterozoic to present day. *Gondwana Res.* **67**, (2019).
