# Supplementary material for: A tighter constraint on Earth-system sensitivity from long-term temperature and carbon-cycle observations
Source: Nat Commun. 2021 May 26;12:3173. doi: 10.1038/s41467-021-23543-9 (PMC8154887; doi:10.1038/s41467-021-23543-9)
Supplement: Supplementary file 3 — Description of Additional Supplementary Files [file 41467_2021_23543_MOESM3_ESM.pdf]

Description of Additional Supplementary Files

File Name: Supplementary Data 1

Description: Table of GEOCARBSULFvolc model parameter descriptions, a priori ranges, a posteriori ranges, and units.

File Name: Supplementary Data 2

Description: Summary table of Earth-system sensitivity estimates from this and other paleoclimate studies, including time periods and radiative forcings considered.
